# Advances in exercise-induced vascular adaptation: mechanisms, models, and methods

**DOI:** 10.3389/fbioe.2024.1370234

**Published:** 2024-02-22

**Authors:** Hualing Sun, Yanyan Zhang, Lijun Shi

**Affiliations:** ^1^ Department of Exercise Physiology, Beijing Sport University, Beijing, China; ^2^ Laboratory of Sports Stress and Adaptation of General Administration of Sport, Beijing Sport University, Beijing, China; ^3^ Key Laboratory of Physical Fitness and Exercise, Ministry of Education, Beijing Sport University, Beijing, China

**Keywords:** exercise, vascular adaptation, hemodynamic stimuli, *in vitro* vascular model, *ex vivo* vascular model

## Abstract

Insufficient physical activity poses a significant risk factor for cardiovascular diseases. Exercise plays a crucial role in influencing the vascular system and is essential for maintaining vascular health. Hemodynamic stimuli generated by exercise, such as shear stress and circumferential stress, directly impact vascular structure and function, resulting in adaptive changes. In clinical settings, incorporating appropriate exercise interventions has become a powerful supplementary approach for treating and rehabilitating various cardiovascular conditions. However, existing models for studying exercise-induced vascular adaptation primarily rely on *in vivo* animal and *in vitro* cellular models, each with its inherent limitations. In contrast, human research faces challenges in conducting mechanistic analyses due to ethics issues. Therefore, it is imperative to develop highly biomimetic *in vitro/ex vivo* vascular models that can replicate exercise stimuli in human systems. Utilizing various vascular assessment techniques is also crucial to comprehensively evaluate the effects of exercise on the vasculature and uncover the molecular mechanisms that promote vascular health. This article reviews the hemodynamic mechanisms that underlie exercise-induced vascular adaptation. It explores the advancements in current vascular models and measurement techniques, while addressing their future development and challenges. The overarching goal is to unravel the molecular mechanisms that drive the positive effects of exercise on the cardiovascular system. By providing a scientific rationale and offering novel perspectives, the aim is to contribute to the formulation of precise cardiovascular rehabilitation exercise prescriptions.

## 1 Introduction

The cardiovascular system is the dynamic organ of the body’s blood circulation, ensuring the relative stability of the internal environment and the normal metabolism. Cardiovascular diseases (CVD) are the most common non-communicable diseases globally, accounting for about one-third of all global deaths (Global Cardiovascular Risk Consortium et al., 2023). Vascular diseases arising from arterial dysfunction are also increasingly attracting international attention. Lack of physical activity is a major risk factor for cardiovascular diseases. In contrast, regular physical activity and exercise training can positively affect the vascular system, improve vascular function, slow down vascular aging, and reduce the risk of vascular-related diseases. Exercise can improve vascular function on one hand and reshape vascular structure (lumen and wall thickness) on the other. In a clinical context, exercise intervention has become an indispensable complementary therapy for treating and rehabilitating various cardiovascular diseases.

Although the importance of exercise for cardiovascular health is recognized, mechanistic analysis in human studies is hindered due to the lack of *ex vivo* vascular models. Existing models for exercise-induced vascular adaptation rely mainly on animal and cell cultures, each with inherent limitations. Animal models suffer from species differences, and two-dimensional cell cultures cannot simulate the complex physiological environment of the body. Therefore, there is an urgent need to establish highly biomimetic *ex vivo* vascular models that simulate exercise stimuli in the human vascular system to elucidate the complex mechanisms underlying the health benefits of exercise.

In recent years, emerging technologies in medicine and life sciences have offered potential solutions to this problem. Various *ex vivo* models such as microfluidics, organ-on-a-chip, bioprinting, computational models, and tissue-engineered blood vessels can more accurately reflect the physiological environment in the body. Combining these models with various vascular assessment techniques is crucial for a comprehensive assessment of the effects of exercise on the vascular system and the intricate molecular mechanisms promoting vascular health.

This article aims to review the hemodynamic mechanisms underlying exercise-induced vascular adaptation. We will explore the limitations of current models for exercise-induced vascular adaptation and the latest advancements in emerging technologies in medicine and life sciences. Based on this, we will evaluate models and methods relevant to exercise-induced vascular adaptation, discussing their future development and challenges. The goal is to provide a scientific basis for revealing the molecular mechanisms behind the beneficial effects of exercise on cardiovascular health and offer new perspectives for formulating more precise vascular rehabilitation exercise prescriptions.

## 2 Exercise-induced vascular adaptation and its hemodynamic mechanisms

**FIGURE 1:**
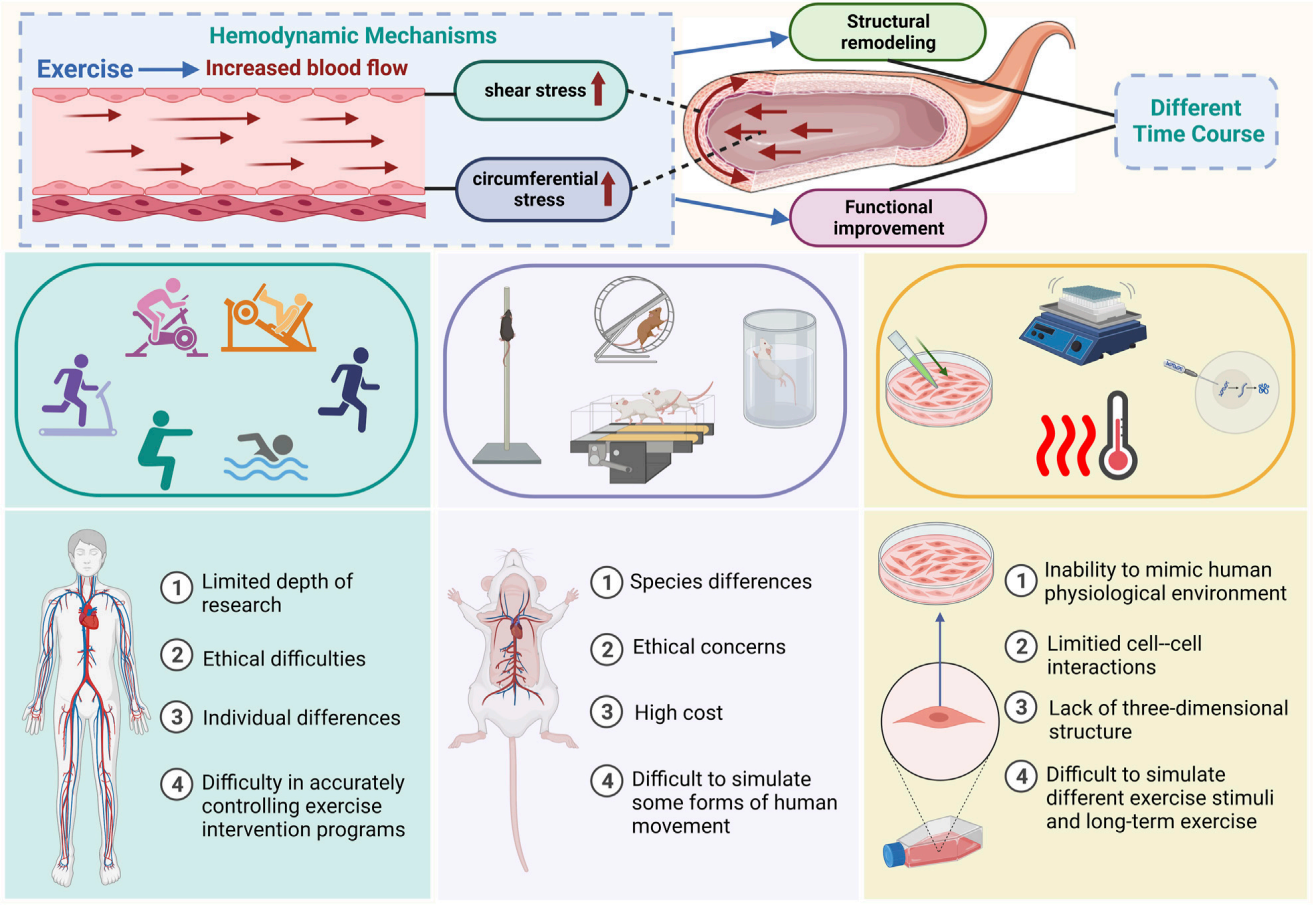
Summary of the hemodynamic mechanisms of exercise-induced vascular adaptation and existing research models and methods The top of the illustration shows the hemodynamic mechanisms of exercise-induced vascular adaptation and the time-course differences between functional and structural adaptation. The middle and bottom sections of the figure illustrate methods of mimicking exercise stimuli in current models (human animal cell) used for studying exercise-induced vascular adaptation and their limitations.

Significant evidence has emerged in recent years suggesting that exercise causes changes to the structure and function of the arteries. Regular exercise positively affects vascular dilatation, vasoconstriction, and compliance; this effect appears stronger in people with poorer vascular function. At the same time, long-term activity, particularly aerobic exercise, causes the artery lumen diameter to increase and the wall thickness to decrease. This structural remodeling, which mostly appears in body parts more actively involved in exercise, is a localized adaptive modification instead of a systemic alteration. Research on humans and animals has shown that exercise can quickly improve vascular dilatation, with short-term exercise increasing nitric oxide (NO) and endothelial nitric oxide synthase (eNOS) production. Training-induced increases in vascular mitochondrial respiratory capacity, as well as evidence of enhanced redox balance, which may be due to increased NO bioavailability, have the potential to protect arterial function from age- and disease-related problems (Park et al., 2016). However, with continuous exercise training, the adaptive structure change will counteract the increase of shear stress and the change in blood vessel function initially caused by exercise. This will result in a different time course between the two events, but the mechanism needs to be clarified (Figure 1).

Blood causes hemodynamic stimuli in the vascular system, and exercise changes blood flow and arterial pressure, causing changes in hemodynamic variables. This eventually leads to adaptive changes in arterial function and structure, altering vascular health. Exercise generates primarily shear stress and circumferential stress as hemodynamic stimulation. Exercise-induced hemodynamic stimuli mainly consist of shear stress and circumferential stress. Shear stress refers to the frictional force acting on the inner surface of blood vessels during blood flow, directly stimulating the endothelium with hemodynamic effects. Circumferential stress is the stress created by the vessel wall in the circular direction, primarily induced by the circumferential movement of blood and the reactive force from the vessel wall. Endothelial and smooth muscle cells can convert mechanical stimuli into chemical signals, impacting ion channels on the cell surface, changing transcription factors and related gene activity, and inducing adaptive modifications (Green et al., 2017).

In endothelial cells, exercise-induced shear stress stimulates the release of many vasodilators, which affect the vascular cells. Components on the endothelial surface, such as the glycocalyx, primary cilia, ion channels, cell membrane receptors, caveolae, and G-proteins, can mechanically transduce shear stress, increasing eNOS and releasing NO. The endothelial cell cytoskeleton and integrins can also connect to the mechanical sensing complex, affecting endothelial cell function (Ando and Yamamoto, 2013). The automatic sensing system of endothelial cells can also translate circumferential stress mechanical stimuli into chemical signals, thereby altering the activation of transcription factors and related genes, including metalloproteinases and growth factors (Kim et al., 2016). Data from *in vitro* cell experiments indicate that circumferential stress produces an anti-atherosclerotic endothelial cell phenotype by upregulating eNOS at the protein and mRNA levels (Dessalles et al., 2021). However, some studies show that circumferential stress reduces the expression of eNOS in endothelial cells (Nikmanesh et al., 2019), promoting atherosclerosis-related cellular features like monocyte chemoattractant protein (MCP)-1 (Hao et al., 2019), intracellular adhesion molecule (ICAM)-1 (Fang et al., 2019), and reactive oxygen species (ROS) (Chang et al., 2019). The contradictory results might be attributed to variations in circumferential stress magnitude and differences in the subjects.

In vascular smooth muscle cells, repetitive shear stress generated by exercise can induce adaptive changes by activating ion channels such as voltage-gated L-type Ca^2+^ channel (Ca_v_1.2), large conductance Ca^2+^-activated K^+^ channel (BK_Ca_), and voltage-gated K^+^ channel (K_V_), thereby altering calcium ion distribution and concentration to regulate the contraction and relaxation of smooth muscle cells (Mokelke et al., 2022). Ca_v_1.2 mediates increased calcium influx, leading to the expression of smooth muscle differentiation-related genes (such as myosin heavy chain, α-actin) and transcription factors (such as cardiomyosin and fibroblast) (Liu et al., 2022). Additionally, circumferential stress increases the production of MCP-1 and O_2_^−^ (Guest et al., 2006; Liu et al., 2022) in smooth muscle cells. It can also affect smooth muscle proliferation and differentiation by regulating connective tissue growth factors, collagen, and fibronectin (Tomoto et al., 2021). Some results show contradictory outcomes between endothelial and smooth muscle cells, possibly due to crosstalk occurring during the response to circumferential stress in these two cell types. Future research may benefit from co-culturing these two cell types for a more comprehensive understanding. Moreover, it is challenging to solely detect the effects of repeated increases in circumferential stress and blood pressure on the vascular system in the human body, as changes in pressure are often correlated with alterations in blood flow and shear stress. This could be a crucial reason why the impact of circumferential stress has not been elucidated in human studies.

## 3 The establishment of the exercise vascular adaptation models and methods

Data on exercise-induced vascular adaptations primarily originate from human studies, animal models, and cell experiments. While research outcomes have demonstrated that exercise can influence vascular structure and function, the specific hemodynamic mechanisms and temporal differences remain unclear. This ambiguity may be associated with limitations in existing models and challenges in establishing a comprehensive platform for integrated vascular model studies. Figure 1 provides an overview of the main existing models for studying exercise-induced vascular adaptation.

### 3.1 Human research

Research on exercise-induced vascular adaptation in humans currently focuses on variations in exercise types or intensities, intervention durations, age groups, and patient populations. Examples include differences in the impact of aerobic exercise, resistance exercise, and high-intensity interval training on blood vessels; distinctions in the effects of low, moderate, and high intensities on blood vessels; variations in the impact of acute and chronic exercise on blood vessels; and differences in the effects of exercise on blood vessels among adolescents, older people, and individuals with medical conditions. Key methods for human research on exercise-induced vascular adaptation include Doppler ultrasound for observing vascular structure and blood flow velocity, magnetic resonance angiography (MRA) for high-resolution imaging of blood vessels, pulse wave velocity (PWV) for assessing vascular elasticity and stiffness, flow-mediated dilation (FMD) for evaluating endothelium-dependent dilation function, and analysis of biomarkers in the blood such as C-reactive protein and NO to assess levels of vascular inflammation and oxidative stress.

While the beneficial effects of exercise on vascular health have been widely acknowledged in human research, the mechanisms underlying the promotion of vascular health by exercise still need to be clarified. The clinical applications are primarily limited to the rehabilitation stage, lacking precise and personalized exercise prescription guidance, remaining in the early stages of development. Human research faces limitations in exploring mechanistic aspects due to the inability to establish *ex vivo* vascular models. The main limitations include: 1. Some methods that allow in-depth study of the internal human vasculature, such as invasive surgery, present ethical and technical difficulties, potential experimental risks, and restrict the depth of research. 2. Human research involves extensive individual variations, including genetic factors, lifestyle, diet, metabolic status, and health conditions, making comparisons between individuals and drawing general conclusions more complex. 3. Various factors influence physical activity, including lifestyle, social environment, and individual choices. Controlling these factors, as well as the type, intensity, and frequency of exercise precisely and completely is challenging in human research. Therefore, human research faces many technical and ethical challenges although it is closer to physiological conditions. Since human studies are more oriented towards observation than well-designed experimentation, researchers need to be careful in experiment design and result interpretation. The optimal approach may involve combining human research with *in vitro* models and clinical studies to better understand exercise-induced vascular adaptation.

### 3.2 Animal models

In exercise-induced vascular adaptation research, most *in vivo* studies utilize animal models as the primary platforms to explore hemodynamic mechanisms. Commonly used experimental animal models include rats, mice, rabbits, pigs, dogs, and non-human primates, with the rodent models being the most widely used in contemporary sport science. The rodent models are extensively employed to investigate the fundamental physiological changes and disease prevention mechanisms induced by exercise interventions in the vascular system. Various research studies typically use exercise interventions such as treadmill exercise, wheel running, swimming, resistance training, etc. (Guo et al., 2020; Wang et al., 2020) to simulate aerobic exercise, resistance training, and multimodal exercises in humans. Researchers also utilize mouse models of hypertension, atherosclerosis, diabetes, etc., to mimic human disease progression and study exercise interventions’ effects on the vascular system.

Despite providing convenient resources for studying the impact of exercise on the vasculature and offering possibilities for exploring the molecular mechanisms of exercise-induced vascular adaptation, animal models still have significant limitations. First, due to species differences, animal models cannot precisely reflect the entire picture of onset and progression when human experience specific diseases. Second, animals cannot fully replicate certain forms of human movement, leading to uncertainties in research results. Third, most animal models have relatively short lifecycles, and their lifespan and exercise parameters do not match those of humans (Feng et al., 2020). Fourth, animal models cannot fully replicate the complex vascular system of humans, especially at the microvascular level. Therefore, future studies should incorporate other *in vitro* models to better translate animal model data and explore molecular mechanisms extensively. Furthermore, establishing an effective humanized blood vessel model will play an increasingly important role as a preclinical model for human research (Driscoll, 2022).

### 3.3 Cell experiments

Although cell culture allows for a more in-depth understanding of the molecular and cellular mechanisms involved in exercise-induced vascular adaptation compared to animal experiments, *in vitro* cell culture induces chemical and physical changes that cannot entirely reflect the complex physiological and biochemical processes influenced by exercise within the body’s blood vessels (Khachigian et al., 2022). Cell models for studying exercise-induced vascular adaptation have limitations. First, the vascular system consists of various cell types that continually interact with each other. Cell experiments often restrict the study to a specific isolated cell type, potentially oversimplifying the complex process of exercise-induced vascular adaptation. Second, current methods for simulating exercise stimuli in cell experiments include drug intervention, mechanical strain, vibration, shear stress, exercise mimetics, cyclic strain, temperature fluctuations, and biomaterials simulating exercise loads. However, there currently lacks a consistent and comprehensive standard for mimicking exercise stimuli using different methods. Consequently, it is challenging to accurately simulate the whole picture of hemodynamic effects induced by exercise. Third, cell models are typically conducted under *in vitro* culture conditions, making it difficult to faithfully reflect the complex environment and three-dimensional tissue structure within the body, including the effects of blood flow, the immune system, and metabolic regulation on blood vessels. Fourth, cell models can barely simulate the complex metabolic states and adaptive changes in the vascular system induced by long-term exercises. Therefore, to better replicate the dynamic complexity of exercise-induced vascular adaptation, there is an urgent need for more advanced *in vitro* vascular models that integrate multiple cell types and more closely simulate physiological conditions. This approach would provide a more comprehensive and physiologically relevant method to study the intricate interactions of different cell types within the vascular system under the influence of exercise.

## 4 Advanced *in vitro* vascular models and research techniques

Significant progress has been made in technologies such as microfluidics, organ-on-a-chip, bioprinting, computational models, and tissue-engineered blood vessels in recent years. Various models of the vascular system have been developed to different extents, allowing for a more accurate representation of physiological environments *in vitro*. These advanced models and technologies in the medical and life sciences fields have the potential to address the limitations of human studies, animal models, and cell-based models in the field of exercise. They are poised to provide more powerful tools and platforms for research on exercise-induced vascular adaptations.

### 4.1 Microfluidics

Microfluidics is a technology that utilizes microchannels and microdevices to manipulate small volumes of liquid precisely. Operating at scales ranging from milliliters to nanoliters, this technique employs tiny structures to control the flow of fluids precisely. Microfluidic devices combine the advantages of traditional *in vitro* and *in vivo* models, such as simplicity, ease of operation, and cost-effectiveness, with the benefits of small-scale operations. Over the past few decades, microfluidics has been widely used for *in vitro* modeling of the vascular system (Simitian et al., 2022). Microfluidics technology can simulate microflow conditions in the vascular system, employing microchannels and microstructures to mimic physiological and pathological conditions of blood vessels. This enables a better understanding of the functionality of the vascular system, disease mechanisms, and the effects of drugs in a vascular environment. Blood vessel microfluidic systems typically include microchannels and microfluidic devices that can simulate blood flow within vessels, interactions between the vessel wall and blood, and the state of vascular cells under different conditions. The technology is extensively applied in researching physiological and pathological processes related to the vascular system, such as cardiovascular diseases, blood clotting, and vascular neogenesis. It provides a more comprehensive approach to studying the complex interactions among different cell types within the vascular system.

Compared to traditional cell culture platforms, microfluidic devices possess unique characteristics in manipulating fluids at a small scale, where highly predictable microscale physics, such as capillary action and laminar flow, dominate over classical macroscopic physics like gravity. This gives users a high degree of controllability and customization in device operation, allowing the integration of various environmental factors that play roles in biological processes, including different cell types and biophysical or biochemical cues (Ayuso et al., 2021). Another advantage of microfluidics is the ability to assess functional readouts, enabling the direct evaluation of cell responses to specific microenvironmental stimuli or treatments, such as cell migration and cell-cell interactions (Ayuso et al., 2022). Microscale physics offers additional advantages for reproducing organ physiology in vascular models, providing precise control over parameters like distance, geometric shape, mechanical cues, and tissue structure (Ayuso et al., 2021) and bridging the gap between traditional 2D and 3D models. Additionally, the small volume of these devices reduces the amount of expensive reagents (cell factors, drugs, scaffold proteins, e.g.) and precious biological samples (such as patient-derived cells or circulating tumor cells) required for each assay. In comparison to traditional *in vitro* models, microfluidic devices, with these advantages, stand out as an ideal platform for conducting *in vitro* vascular research.

### 4.2 Organ-on-a-chip

“Organ-on-a-Chip” is a biomedical engineering technology that utilizes principles of microfluidics, microfabrication, and bioengineering to simulate the structure and function of human organs on a small chip. Over the past decade, Organ-on-a-Chip has rapidly developed, becoming an alternative method for crucial *in vitro* modeling in life sciences research (Ingber, 2022). The Organ-on-a-Chip technology provides a unique platform for studying the vascular system, allowing researchers to simulate the physiological and pathological processes of the human blood vessels more accurately. This technology enables the design of microvascular networks that mimic real arteries, veins, and capillaries. These microchannels can be infused with simulated blood, allowing the researchers to observe and analyze hemodynamics, vascular wall responses, and cellular behaviors within the blood vessels. *In vitro* organ models can incorporate various cell types, including endothelial cells, smooth muscle cells, and other relevant cells, to simulate cellular interactions within real vascular tissues. This aids in a better understanding of biological processes within blood vessels, such as vascular wall growth, maintenance, and repair.

Using Organ-on-a-Chip, researchers can establish models for various vascular diseases, such as atherosclerosis, hypertension, thrombosis, etc. By introducing specific pathological conditions, researchers can study the mechanisms of disease development and evaluate potential treatment methods. For example, organoids derived from human stem cells faithfully recapitulate the structure and function of human blood vessels and are amenable systems for modelling and identifying the regulators of diabetic vasculopathy (Wimmer et al., 2019). Additionally, this technology can be used for drug screening and assessment, contributing to increased efficiency in drug development and reducing the need for animal experiments. Organ-on-a-chip allows for a better understanding of the interactions between blood and blood vessels, facilitating blood-vessel interface research and simulating the microenvironment within blood vessels, including platelet aggregation and vascular permeability (Wang et al., 2023). Organ-on-a-chip can also establish personalized medical models, making research more targeted and considering individual differences. Overall, the application of Organ-on-a-Chip in the vascular system provides researchers with a highly controllable experimental platform, potentially advancing research on the vascular system and the development of treatment methods for related diseases.

### 4.3 Bioprinting

Bioprinting refers to utilizing computer-aided design (CAD) models to construct three-dimensional biological tissues or organs by adding natural materials or cells layer-by-layer according to predetermined structures and shapes. This technology can simulate and reconstruct the complex structures of the human vascular system, providing support for tissue engineering, organ transplantation, and other related applications. 3D bioprinting has improved control over vascular growth, repeatability, and the scalability of the manufacturing process, becoming a crucial tool in the production of vascularized biological constructs (Richards et al., 2017). Additionally, the latest 4D bioprinting technology integrates time-sensitive components into the bioprinting process, enhancing the responsiveness of constructs to environmental stimuli and improving the formation of realistic vascularized tissues (Zhou et al., 2020). In recent years, bioprinting technology has significantly progressed in forming vascular tissues or constructing vascular network structures. Researchers in tissue engineering, materials science, and medicine have developed a range of unique printing processes and biocompatible materials to optimize the mechanical performance, low cytotoxicity, biodegradability, and biocompatibility of this technology. Furthermore, advancements in bioprinting technology enable the layer-by-layer printing of cell aggregates, tissue strands, or biomaterials loaded with cells and growth factors based on predefined shapes created using CAD software.

In vascular bioprinting, bioprinters construct biological tissues layer by layer by stacking natural materials and cells according to predetermined vascular structures and shapes. These biological materials typically include cell-compatible scaffold materials (bioink) and cells to ensure cell viability and functionality during printing (Kong and Wang, 2023). After printing is completed, further processing can be carried out, such as cross-linking scaffold materials to enhance structural stability and adding bioactive substances to promote the normal physiological function of blood vessels. Bioprinting technology opens new possibilities for organ transplantation, tissue engineering, drug screening, disease research, and personalized medicine in the vascular system. This technology is poised to bring more innovation and advancements to medicine and biomedical research by simulating and reconstructing complex vascular structures.

### 4.4 Computational models

A computational model uses computer simulation and modeling techniques, employing numerical calculation methods such as mathematical and physical equations, to construct models on a computer for simulating and analyzing physiological and pathological processes related to specific systems or organs within a biological organism. In the biomedical field, *in silico* computational models span multiple levels, including the molecular, cellular, tissue, and organ levels. Typically based on a deep understanding of relevant processes within the biological organism, these models use mathematical modeling and simulation to mimic interactions and dynamic behaviors within biological systems. They study the emotional behaviors, response mechanisms, disease development, etc., within biological systems. Computational models find extensive application in vascular system research, where they can simulate aspects such as blood flow dynamics, vascular wall reactions, and disease mechanisms, providing crucial support for disease research, drug development, and treatment strategy design (Corti et al., 2021). They are utilized in various aspects such as hemodynamics simulation, vascular wall reaction simulation, vascular pharmacology research, vascular pathology research, surgical planning and simulation, and personalized medicine. They contribute essential theoretical and experimental foundations to disease diagnosis, treatment strategy formulation, and basic scientific research.

### 4.5 Tissue-engineered blood vessels

Tissue-engineered blood vessels refer to applying principles and techniques of tissue engineering to cultivate and construct functional vascular structures *in vitro*. This technology aims to generate novel tissues that can be used to repair, replace, or enhance damaged blood vessels. Tissue-engineered blood vessels typically involve the use of components such as biomaterials, bioactive molecules, and cells. Through suitable scaffolds or support structures, they promote the growth and development of vascular tissue under *in vitro* culture conditions. The ultimate objective of tissue-engineered blood vessels is to produce vascular structures with physiological functionality, improving the supply of tissue-engineered transplant vessels for treating cardiovascular diseases (Moore et al., 2022). They find applications in various fields, including physiological and pathological research, drug screening and evaluation, transplantation and repair, biomaterial research, cell interaction studies, and personalized medicine. This technology holds potential clinical and research applications for addressing vascular defects and improving the survival and function of tissue-engineered organs. It provides researchers with a highly controllable experimental platform, offering prospects for advancing vascular system research and developing related disease treatments.

## 5 Perspective—applying advanced models and methods to the field of sports

**FIGURE 2:**
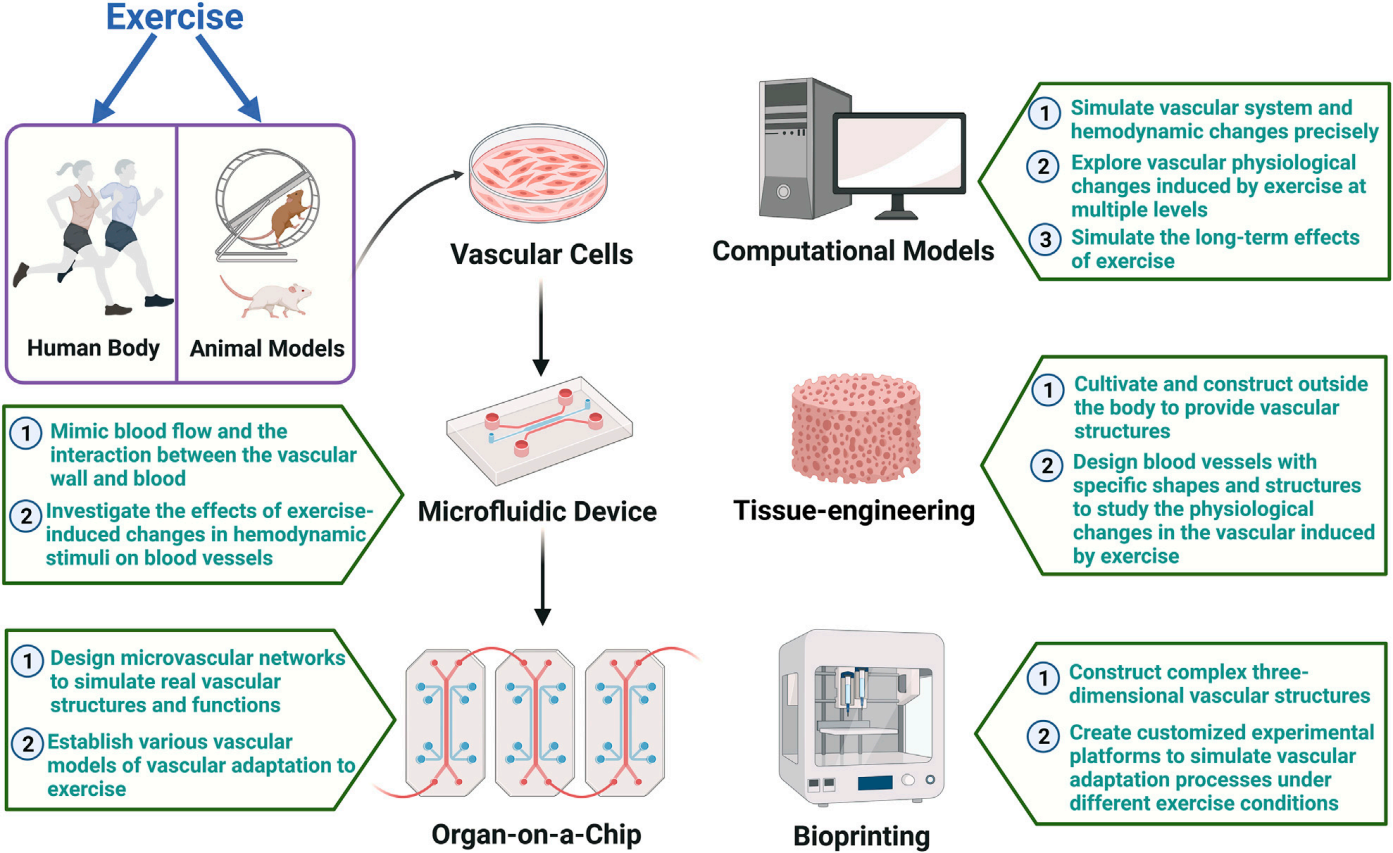
Summary of how to apply advanced vascular modeling in sports science The figure illustrates how emerging technologies such as microfluidics, organ-on-a-chip, tissue-engineered, computational models, and bioprinting blood vessels can be integrated with existing techniques and applied in the field of exercise. The establishment of advanced vascular models on highly sophisticated experimental platforms will facilitate in-depth research into the effects of exercise on the vascular system and the molecular mechanisms promoting vascular health.

In the future, applying emerging technologies such as Microfluidics, Organ-on-a-Chip, Bioprinting, Computational Models, and Tissue-Engineered Blood Vessels to the field of exercise can establish highly advanced experimental platforms for in-depth research on the effects of exercise on the vascular system and the molecular mechanisms promoting vascular health. Figure 2 provides a summary of how to apply advanced vascular modeling in sports science in the future. Here is an overview of the potential applications of each technology:

*Microfluidics:* Precise manipulation at the microscale can address the challenges of in-depth human studies. Simulating microflows within the vascular system using microfluidics aids in understanding the impact of hemodynamic stimuli on the microenvironment of blood vessels. Microfluidic devices can mimic blood flow and the interaction between the vascular wall and blood, providing a more accurate *in vitro* model for studying vascular adaptation to different exercise stimuli with high controllability. By integrating flexible and stretchable electrochemical sensor into microfluidic vascular chips, studies have been conducted to apply cyclic circumferential stretch to endothelial cells while monitoring mechanically-induced biochemical signals (Jin et al., 2020). This opens the possibility of using microfluidic technology to investigate the effects of exercise-induced changes in circumferential stress on blood vessels.

*Organ-on-a-Chip:* By designing microvascular networks, Organ-on-a-Chip technology can simulate real vascular structures and functions. It overcomes the limitations of dynamic three-dimensional structure studies in animal and cell models (Mandrycky et al., 2021). This technology can establish various vascular models by introducing different populations (such as middle-aged individuals, seniors, and athletes of different sports) or specific pathological conditions (such as hypertension and atherosclerosis), allowing targeted studies on vascular adaptation to exercise and addressing the deficiencies of existing models in considering individual differences.

*Bioprinting:* Bioprinting can construct complex three-dimensional vascular structures, providing a more realistic *in vitro* model for studying exercise-induced vascular adaptation. The high controllability of this technology enables researchers to create customized experimental platforms to simulate vascular adaptation processes under different exercise conditions, offering a comprehensive understanding of the effects of exercise on the structure and function of the vascular system.

*Computational Models:* Computational models use mathematical and physical equations to simulate vascular system and hemodynamic changes precisely. This approach aids in understanding vascular physiological changes induced by exercise at multiple levels and exploring the molecular mechanisms of exercise-induced vascular adaptation. Computational models can also simulate the long-term effects of exercise, providing a more comprehensive perspective on adaptive changes in the vascular system compared to current human, animal, and cell models.

*Tissue-Engineered Blood Vessels:* Tissue-Engineered Blood Vessels can be cultivated and constructed outside the body, providing functional vascular structures for a more realistic *in vitro* model. This technology allows the study of physiological changes in the vascular induced by exercise, including vascular elasticity, endothelial function, and smooth muscle cells, addressing limitations in structure and function seen in animal and cell models. Engineered blood vessels can be designed with specific shapes and structures, including vessel diameter, wall thickness, and branching patterns, aiding in revealing details of exercise-induced vascular adaptation.

By integrating the above technologies, a more comprehensive, accurate, and controllable experimental platform can be established to study exercise-induced vascular adaptation at a broader level. This approach overcomes the limitations of current research models and methods, providing a more holistic understanding of the effects of exercise on the vascular system. It unravels the complex molecular mechanisms through which exercise promotes vascular health, offering new scientific insights and perspectives for developing more precise exercise prescriptions for vascular rehabilitation.
